# Effectiveness of Seasonal Influenza Vaccine against Pandemic (H1N1) 2009 Virus, Australia, 2010

**DOI:** 10.3201/eid1707.101959

**Published:** 2011-07

**Authors:** James E. Fielding, Kristina A. Grant, Katherine Garcia, Heath A. Kelly

**Affiliations:** Author affiliations: Victorian Infectious Diseases Reference Laboratory, North Melbourne, Victoria, Australia (J.E. Fielding, K.A. Grant, K. Garcia, H.A. Kelly);; The Australian National University, Canberra, Australian Capital Territory, Australia (J.E. Fielding)

## Abstract

To estimate effectiveness of seasonal trivalent and monovalent influenza vaccines against pandemic influenza A (H1N1) 2009 virus, we conducted a test-negative case–control study in Victoria, Australia, in 2010. Patients seen for influenza-like illness by general practitioners in a sentinel surveillance network during 2010 were tested for influenza; vaccination status was recorded. Case-patients had positive PCRs for pandemic (H1N1) 2009 virus, and controls had negative influenza test results. Of 319 eligible patients, test results for 139 (44%) were pandemic (H1N1) 2009 virus positive. Adjusted effectiveness of seasonal vaccine against pandemic (H1N1) 2009 virus was 79% (95% confidence interval 33%–93%); effectiveness of monovalent vaccine was 47% and not statistically significant. Vaccine effectiveness was higher among adults. Despite some limitations, this study indicates that the first seasonal trivalent influenza vaccine to include the pandemic (H1N1) 2009 virus strain provided significant protection against laboratory-confirmed pandemic (H1N1) 2009 infection.

After the emergence and rapid global spread of pandemic influenza A (H1N1) 2009 virus, development of a pandemic (H1N1) 2009–specific vaccine began (*1*). A candidate reassortant vaccine virus, derived from the A/California/7/2009 (H1N1)v virus as recommended by the World Health Organization, was used to produce a monovalent, unadjuvanted, inactivated, split-virus vaccine for Australia (*2*,*3*). The national monovalent pandemic (H1N1) 2009 vaccination program in Australia ran from September 30, 2009, through December 31, 2010, and vaccination was publicly funded for all persons in Australia >6 months of age (*4*,*5*).

In September 2009, the World Health Organization recommended that trivalent influenza vaccines for use in the 2010 influenza season (Southern Hemisphere winter) contain A/California/7/2009 (H1N1)–like virus, A/Perth/16/2009 (H3N2)–like virus, and B/Brisbane/60/2008 (of the B/Victoria/2/87 lineage) virus (*6*). Since March 2010, the Australian Government has provided free seasonal influenza vaccination to all Australia residents >65 years of age, all Aboriginal and Torres Strait Islander persons >50 years, all Aboriginal and Torres Strait Islander persons 15–49 years with medical risk factors, persons >6 months with conditions that predispose them to severe influenza, and pregnant women (*7*). Influenza vaccination is also recommended, but not funded, for persons who might transmit influenza to those at high risk for complications from influenza, persons who provide essential services, travelers, and anyone >6 months of age for whom reducing the likelihood of becoming ill with influenza is desired. Individual industries are also advised to consider the benefits of offering influenza vaccine in the workplace (*8*). Because pandemic (H1N1) 2009 was expected to be the dominant strain in 2010, the monovalent vaccine continued to be used despite the availability of the seasonal vaccine, particularly by persons who were not eligible for funded vaccine (M. Batchelor, pers. comm.). However, in 2010, there were no published data on the relative use of monovalent and seasonal vaccines at that time.

The need for rapid implementation of programs results in initial studies using immunogenicity, rather than efficacy, to assess performance of influenza vaccines. After 1 dose of monovalent pandemic (H1N1) 2009 vaccine containing 15 µg hemagglutinin without adjuvant, seroprotection was estimated to be 94%–97% in working-age adults (*3*,*9*,*10*) and 75% in children (*10*). Observational studies provide a practical way to calculate vaccine effectiveness under field conditions (*11*,*12*). Effectiveness of monovalent pandemic (H1N1) 2009 was estimated to be 72%–97% by 3 studies in general practice and community-based settings in Europe (*13*–*15*), 90% in a hospital-based study in Spain (*16*), and 100% in a community-based study of children in Canada (*17*). These studies were conducted in populations for which the respective local or national pandemic vaccination program primarily used vaccine without adjuvant.

We assessed effectiveness of the 2010 seasonal influenza vaccine against laboratory-confirmed pandemic (H1N1) 2009 influenza infection in Victoria, Australia. Data came from an established test-negative case–control study in a general practitioner sentinel surveillance network (*18*,*19*).

## Methods

### Sentinel Surveillance

Victoria is the second most populous state in Australia; it has a temperate climate, and the annual influenza season usually occurs during May–September. Each season, on behalf of the Victorian Government Department of Health, the Victorian Infectious Diseases Reference Laboratory conducts surveillance for influenza-like illness (ILI; defined as history of fever, cough, and fatigue/malaise) and laboratory-confirmed influenza. General practitioners within the network provide weekly reports on case-patients with ILI as a proportion of total patients seen and send swabs from patients with ILI to the laboratory for testing. In 2010, a total of 87 practitioners participated in the program, which operated for 25 weeks, from May 3 (week 19) through October 24 (week 43). Practitioners were asked to collect nose and throat swabs from patients with an ILI (*20*) within 4 days after onset of the patient's symptoms. Samples were collected by using Copan dry swabs (Copan Italia, Brescia, Italy) and were placed in virus transport medium. Practitioners were also asked to provide data on the patient's age, sex, date of symptom onset, vaccination status, type of influenza vaccine (monovalent or trivalent/seasonal) received, and date of vaccination. Type of vaccine and date of vaccination were ascertained from medical records and patient report.

### Laboratory Testing

RNA was extracted from clinical specimens by using a Corbett extraction robot (Corbett Robotics, Brisbane, Australia), followed by reverse transcription to cDNA by using random hexamers. PCR amplification and detection selective for the type A influenza virus matrix gene was performed by using primers and a Taqman probe on an ABI-7500 Fast Real-Time PCR system (Applied Biosystems, Foster City, CA, USA). Samples determined to be positive by this assay were confirmed as positive or negative for pandemic (H1N1) 2009 in a second real-time PCR that incorporated primers and probes specific for the hemagglutinin gene of the pandemic (H1N1) 2009 virus. Influenza B viruses were identified by a separate PCR. One practitioner chose to send samples to the state reference laboratory in South Australia for testing with equivalent diagnostic assays.

### Ascertainment of Case-patients and Controls

Case-patients and controls were sampled prospectively throughout the study period. A case-patient was defined as a person with ILI for whom test results for pandemic (H1N1) 2009 were positive; a control was defined as a person with negative test results for influenza virus. Analysis of vaccine effectiveness against other influenza subtypes was not undertaken because of the almost exclusive circulation of pandemic (H1N1) 2009 virus during the season; therefore, patients with positive test results for other influenza viruses were excluded. A control could become a case-patient if another illness developed during the season, but a case-patient was no longer at risk and could not be included again.

### Data Analysis and Calculation of Vaccine Effectiveness

All analyses were conducted by using Stata version 10.0 (StataCorp LP, College Station, TX, USA). The χ^2^ test was used to compare proportions, and the Mann-Whitney U test was used to compare time from vaccination to time seen by practitioner; p<0.05 was considered significant. Patients were excluded from the vaccine effectiveness analysis if vaccination status was unknown, if the date of symptom onset was unknown, or if the interval between symptom onset and specimen collection was >4 days (because of decreased likelihood of a positive result after this time) (*21*,*22*). Patients were considered not vaccinated if time between date of vaccination and symptom onset was <14 days. If only the month of vaccination was reported, the date of vaccination was conservatively estimated to be the last day of the month. To avoid overestimation of vaccine effectiveness arising from recruitment of controls when influenza was not circulating in the population, analysis was restricted to case-patients and controls detected within the influenza season, defined as the period during which influenza-positive case-patients were detected (weeks 26–40).

Vaccine effectiveness was defined as (1–odds ratio) × 100%; the odds ratio is the odds of laboratory-confirmed pandemic (H1N1) 2009 case-patients having been vaccinated divided by the odds of controls having been vaccinated. In the test-negative case–control design, the odds ratio estimates the incidence density (rate) ratio because controls are selected longitudinally throughout the course of the study (i.e., by density sampling) (*23*,*24*). The odds ratio in test-negative case–control studies has also been shown to approximate the risk ratio under conditions of varying attack rates and test sensitivity and specificity (*25*). Logistic regression was used to calculate odds ratios and 95% confidence intervals (CIs) for having laboratory-confirmed pandemic (H1N1) 2009, which were adjusted for the variables of age group and month of specimen collection against the following: seasonal vaccine, monovalent vaccine, both vaccines, and any (either or both the seasonal and monovalent) vaccine. Sensitivity analyses were conducted to determine the effects of the following on vaccine effectiveness: not censoring for specimens collected from ILI patients >4 days after symptom onset, including controls recruited outside the defined influenza season, and assuming that patients with unspecified type A influenza had pandemic (H1N1) 2009.

### Ethical Considerations

Data in this study were collected, used and reported under the legislative authorization of the Victorian Public Health and Wellbeing Act 2008 and Public Health and Wellbeing Regulations 2009. Thus, the study did not require Human Research Ethics Committee approval.

## Results

A total of 172,411 patients were seen by participating practitioners during the study period, of whom 678 (0.4%) had ILI. After a nadir ILI rate of 0.2% in week 21, the rate gradually increased to 0.4% in week 31 before increasing more sharply to a peak of 0.9% in week 36. Swabs were collected from 478 (71%) ILI patients, among whom 170 (36%) had positive influenza test results and the remainder were negative. Influenza-positive patients were detected during weeks 26–40, which was defined as the influenza season (Figure). A total of 142 patients were excluded from further analysis because vaccination status was unknown (n = 11), symptom onset date was unknown (n = 33), time between symptom onset and specimen collection was >4 days (n = 43), or the specimen was collected outside the influenza season (n = 82). A significantly higher proportion of influenza-negative patients (13%) than influenza-positive patients (4%) were excluded because >4 days had elapsed between symptom onset and specimen collection (p = 0.001). No significant difference was found by age group for whether study participants had a specimen collected within 4 days after symptom onset (p = 0.10).

**Figure:**
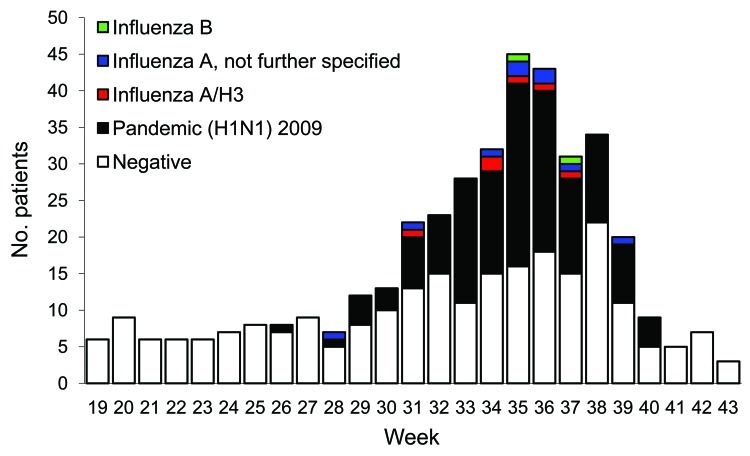
Influenza status of patients seen at sentinel general practices, Victoria, Australia, May 3 (week 19) through October 24 (week 43), 2010.

Of the remaining 336 patients, 156 (46%) had positive influenza test results. Most (89%) influenza case-patients had pandemic (H1N1) 2009, 6% had unspecified type A influenza, 4% had influenza A (H3N2), and 1% had influenza type B (Figure). After exclusion of the other influenza patients, 139 pandemic (H1N1) 2009 case-patients and 180 controls were included in the study analysis. Most (57%) participants were 20–49 years of age, and case-patients were significantly younger than controls (p = 0.001); no case-patient was >65 years of age (Table 1). No statistically significant difference was found between male and female study participants by case or control status (p = 0.60) or by vaccination status (p = 0.09). The high proportion of case-patients detected in August resulted in a significant difference between case-patients and controls by month of swab collection (p<0.001).

**Table 1:** Participants in negative-test case–control study of efficacy of seasonal influenza vaccine for preventing pandemic (H1N1) 2009, Australia, 2010

Participants	Age group, y					Total, n = 319
	0–4, n = 19	5–19, n = 73	20–49, n = 181	50–64, n = 41	>65, n = 5	
Controls						
Total*	13 (68)	27 (37)	107 (59)	28 (68)	5 (100)	180 (56)
Vaccinated with monovalent vaccine†	0	3 (11)	7 (7)	1 (4)	0	11 (6)
Vaccinated with seasonal vaccine†	0	0	9 (8)	10 (36)	2 (40)	21 (12)
Vaccinated with both vaccines†	0	0	7 (7)	4 (14)	2 (40)	13 (7)
Pandemic (H1N1) 2009 case-patients						
Total*	6 (32)	46 (63)	74 (41)	13 (32)	0	139 (44)
Vaccinated with monovalent vaccine†	0	3 (7)	3 (4)	0	0	6 (4)
Vaccinated with seasonal vaccine†	0	2 (4)	2 (3)	0	0	4 (3)
Vaccinated with both vaccines†	0	0	2 (3)	0	0	2 (1)

*No. (%) study participants. †No. (%) controls/pandemic (H1N1) 2009 case-patients.

Overall, 59 (18%) study participants were reported as vaccinated with any vaccine, but the proportion was higher among controls (26%) than among case-patients (9%; p<0.001). The proportion of controls, who were mostly older, who had received the trivalent seasonal vaccine was higher than the proportion of controls who had received the monovalent vaccine (Table 1). Similarly, controls who had received both vaccines were all >20 years of age. Only case-patients who were 5–19 and 20–49 years of age were reported as vaccinated. Influenza vaccine type was not specified for 1 case-patient and 1 control, each of whom was reported as vaccinated.

Reflecting the availability of each vaccine, the median period between vaccination and visit to a general practitioner was significantly shorter for those who received seasonal vaccine (114 days) than for those who received monovalent vaccine (223 days; p<0.0001). No significant difference in the time from vaccination to practitioner visit was found between case-patients and controls for seasonal (p = 0.70) or monovalent vaccine (p = 0.95).

In general, point estimates of vaccine effectiveness adjusted for patient age and month of specimen collection differed little from crude estimates (Table 2). A significant protective effect was observed for seasonal vaccine only (adjusted vaccine effectiveness 79%; 95% CI 33%–93%) and seasonal and monovalent vaccines (adjusted vaccine effectiveness 81%; 95% CI 7%–96%). The adjusted vaccine effectiveness for receipt of any (either or both the seasonal and monovalent) vaccine was lower at 67% because of the 47% vaccine effectiveness for monovalent vaccine. The absence of vaccinated case-patients and controls meant vaccine effectiveness could not be estimated for several of the 5 age groups (Table 1); therefore, age was collapsed into 3 variables: children (0–19 years), working-age adults (20–64 years), and elderly persons (>65 years). Estimates of vaccine effectiveness for working adults were 0%–14% higher than the overall adjusted estimates; estimates for children were either undefined because no controls were vaccinated or were without a significant protective effect. Vaccine effectiveness could not be calculated for elderly persons because there were no case-patients in this age group.

**Table 2:** Crude and adjusted vaccine effectiveness against pandemic (H1N1) 2009 virus, Australia, 2010

Effectiveness	Influenza vaccine effectiveness, % (95% confidence interval)			
	Seasonal	Monovalent	Both	Any
Crude	80 (39–93)	42 (−62 to 79)	84 (26 to 96)	70 (42 to 84)
Adjusted*				
0–19 y	Undefined†	44 (−231 to 91)	Undefined‡	−41 (−549 to 69)
20–64 y	89 (50 to 98)	56 (−88 to 90)	81 (7 to 96)	81 (52 to 92)
All ages	79 (33 to 93)	47 (−62 to 82)	81 (7 to 96)	67 (33 to 84)

*Adjusted for month of swab collection. †No controls vaccinated. ‡No controls or case-patients vaccinated.

Sensitivity analyses to determine the effects of certain assumptions resulted in variations in the adjusted vaccine effectiveness point estimates of 0%–3% and no changes to their relative significance. The effects considered were as follows: assumption that those patients with unspecified influenza type A had pandemic (H1N1) 2009, no exclusion of patients if >4 days had elapsed between symptom onset and specimen collection, and no exclusion of patients if they were identified outside the defined influenza season.

## Discussion

Our results indicate that the 2010 seasonal trivalent influenza vaccine is >80% effective against pandemic (H1N1) 2009 virus, regardless whether given by itself or in addition to monovalent vaccine. Groups in Europe and Canada have estimated the effectiveness of monovalent seasonal influenza vaccine against pandemic (H1N1) 2009 virus to be 72%–100% (*13*–*17*). However, the effectiveness of any vaccine (monovalent, seasonal, or both) against pandemic (H1N1) 2009 virus was lower (67%, 95% CI 33%–84%) because effectiveness for monovalent vaccine only was 47% (95% CI –62% to 82%). The lower effectiveness of monovalent influenza vaccine against pandemic (H1N1) 2009 virus compared with seasonal trivalent influenza vaccine is difficult to explain. Both vaccines contain the same quantities (15 µg) of hemagglutinin; and although the monovalent vaccine does not contain adjuvant and was available ≈6 months before the seasonal vaccine, it has been shown to be strongly immunogenic (*3*,*9*,*10*). Immunogenicity does not necessarily correlate directly with vaccine effectiveness, and we cannot exclude waning immunity as an explanation for the lower effectiveness of monovalent vaccine in our study. Waning immunity after receipt of monovalent vaccine has been suggested after an interim study from the United Kingdom for the 2010–11 influenza season (*26*). The finding could also be a product of the relatively small number of case-patients and controls who received only the monovalent vaccine, given that vaccine effectiveness estimates can change considerably by the inclusion or exclusion of 1–2 vaccinated study participants.

When stratified by age, estimates of vaccine effectiveness for working-age adults were higher and more precise than those for children. We previously demonstrated that the sentinel practitioner surveillance program in Victoria is well suited for estimating vaccine effectiveness among working-age adults, who account for most of the surveillance population (*18*), and the 2010 results were consistent with this observation. The relatively few participants in the young (childhood) age groups meant the study had insufficient power to produce defined or significant estimates of vaccine effectiveness. At the other end of the age spectrum, 2% of study participants (5 controls and 0 case-patients) in 2010 were >65 years of age compared with an average of 7% in this age group during 2003–07 (*18*). Although the absence of pandemic (H1N1) 2009 case-patients >65 years of age is not surprising, given that older adults have been shown to have relatively higher levels of cross-reactive antibodies to pandemic (H1N1) 2009 virus (*27*–*29*), the reason for the low proportion of controls in this age group remains unclear. Among the several explanations are a true lower rate of ILI in older persons during 2010, a lower rate of visits to practitioners for ILI by persons in this age group (or treatment at other health services such as hospitals), or preferential sampling of younger persons by practitioners (and perhaps awareness that pandemic [H1N1] 2009 was the predominant circulating influenza virus subtype).

In addition to having a sample size large enough to provide vaccine effectiveness estimates by age group and influenza type, several other considerations with regard to design of case–control studies of influenza vaccine effectiveness have been proposed: 1) whether the control group best represents the vaccination coverage of the source population and 2) whether collection and confounding variables have been adjusted for, particularly underlying chronic conditions for which vaccine is recommended and previous influenza vaccination history (*30*). A 2010 survey of pandemic vaccination suggests that monovalent vaccine coverage in the control group was generally consistent with that in the general population and that use of monovalent vaccine was ≈17% among those from Victoria, compared with 13% among controls (*31*). No equivalent survey of 2010 seasonal vaccine usage was available for comparison.

Data about concurrent conditions of study participants that would indicate need for influenza vaccination were not collected during the 2010 influenza season; thus, adjustment of the vaccine effectiveness estimates for this potentially confounding variable could not be conducted. Such confounding by indication (or negative confounding), in which persons at higher risk for influenza are more likely to be vaccinated, underestimates effectiveness of influenza vaccine but may be counteracted by healthy vaccinee bias (or positive confounding), which overestimates effectiveness (*30*,*32*). The extent to which these biases occur is likely to vary and may explain the positive and negative variation of crude influenza vaccine effectiveness estimates after adjustment for chronic conditions in several similar test-negative case–control studies (*33*–*35*). Speculation about the relative effects of these biases on how many received monovalent vaccine is also difficult; vaccination was funded for the entire population of Australia, but at the end of February 2010, only 18% had been vaccinated (*31*).

Similar methods using test-negative controls to assess seasonal and pandemic vaccine effectiveness against both seasonal and pandemic influenza viruses have been applied in North America and Europe (*13*,*16*,*17*,*33*–*39*). Observational studies provide a convenient and timely way to assess influenza vaccine effectiveness without the ethical, practical, and financial stringencies associated with clinical trials for vaccine efficacy, but they also have limitations. Modeling suggests that the test-negative case–control design generally underestimates true vaccine effectiveness under most conditions of test sensitivity, specificity, and the ratio of influenza to noninfluenza attack rates (*25*), although quantifying the extent of this effect in this study is difficult because the precise sensitivity and specificity of the test are not known. We attempted to limit ascertainment bias by censoring records that indicated specimen collection >4 days after symptom onset and restricting the analysis to case-patients and controls tested within the influenza season only, although sensitivity analyses indicated little effect if these restrictions were relaxed. Of note, these findings apply predominantly to working-age adults receiving medical care in the general practice setting; the study did not include those who did not seek medical care for ILI. Thus, the study measured effectiveness of vaccine against illness severe enough to require a visit to a practitioner; the results cannot necessarily be generalized to other parts of the population, in particular young children and elderly persons. We were also unable to determine whether participants had previously been infected with pandemic (H1N1) 2009 virus, which may result in overestimation of vaccine effectiveness.

In conclusion, we applied a test-negative case–control study design to an established sentinel surveillance system to estimate effectiveness of a trivalent seasonal influenza vaccine, which included an A/California/7/2009 (H1N1)–like virus, the pandemic (H1N1) 2009 influenza virus strain. This strain is also a component of the trivalent influenza vaccine for the 2010–11 Northern Hemisphere influenza season (*40*). The trivalent vaccine provided significant protection against laboratory-confirmed pandemic (H1N1) 2009 virus infection.
